# Community-Acquired Methicillin-Resistant Staphylococcus Aureus Hepatic Liver Abscess

**DOI:** 10.7759/cureus.12560

**Published:** 2021-01-07

**Authors:** Akwe Nyabera, Lilia Shaban, Kristin Hijazin, Taisiya Tumarinson

**Affiliations:** 1 Internal Medicine, Icahn School of Medicine at Mount Sinai, Queens Hospital Center, New York, USA; 2 Medicine, St. George's University School of Medicine, St. George's, GRD; 3 Medicine, Icahn School of Medicine at Mount Sinai, Queens Hospital Center, New York, USA

**Keywords:** methicillin resistant staphylococcus aureus (mrsa), liver abscess drainage

## Abstract

A methicillin-resistant Staphylococcus aureus (MRSA) liver abscess is a rare infection that if not recognized, and treated early, can be fatal. There is limited literature demonstrating possible etiologies of MRSA liver abscesses, whether nosocomial or community acquired. We present a case of a 45-year-old Guyanese male with a 30 pack-year smoking history. The patient presented with both generalized abdominal pain and a productive cough on two separate occasions. Laboratory results in his second presentation revealed leukocytosis with increased alanine transaminase (ALT). Imaging revealed a multiloculated abscess in the inferior aspect of the liver, measuring 5.1 cm x 3.4 cm x 4 cm, and chest X-ray revealed developing consolidation within the right perihilar region. The patient underwent percutaneous liver drainage via pigtail catheter. Fluid cultures grew MRSA. The patient was placed on vancomycin for three weeks. On subsequent examinations, there was a resolution of leukocytosis with no drainage from the pigtail catheter. Elevations of aspartate transaminase (AST), ALT, and gamma-glutamyl transferase (GGT) were observed. Therefore, in addition to restarting vancomycin, the patient was started on ciprofloxacin for two weeks and liver function tests (LFTs) trended downwards, without recurrence of symptoms. High suspicion for liver abscess should exist in patients that present with complaints of abdominal pain and elevated LFTs when a previous source of infection has been observed. MRSA liver abscesses are rare and potentially fatal, therefore, early recognition and appropriate management is essential.

## Introduction

A liver abscess is defined as a pus-filled collection that is caused by factors such as abdominal infections, trauma to the liver, infections in the biliary duct, and hematogenous spread from other locations. Liver abscesses can be categorized as either pyogenic or amoebic [1]. In 50% of infections, the abscess will form in the right lobe of the liver due to the anatomical insertion of the hepatic and portal arteries [1,2]. Risk factors for abscess formation include diabetes, cirrhosis, male gender, older age, immunocompromised states, and the use of proton pump inhibitors [2]. Pyogenic liver abscesses have an incidence of 0.5%-0.8%, with only 10% of these abscesses caused by Staphylococcus aureus, and even fewer by methicillin-resistant Staphylococcus aureus (MRSA) [3]. Approximately 13 cases of MRSA liver abscesses have been reported in the English literature, with only five being community acquired [3]. Once a liver abscess has been diagnosed, therapeutic intervention includes empiric antibiotic administration and percutaneous drainage with microbiological assay [4]. Empiric antibiotic treatment involves broad-spectrum antibiotics [5]. Failure to recognize the infection and achieve timely management may lead to a poor prognosis, with possible sequelae including sepsis, peritonitis, or empyema [1,6] 

## Case presentation

**Figure 1:**
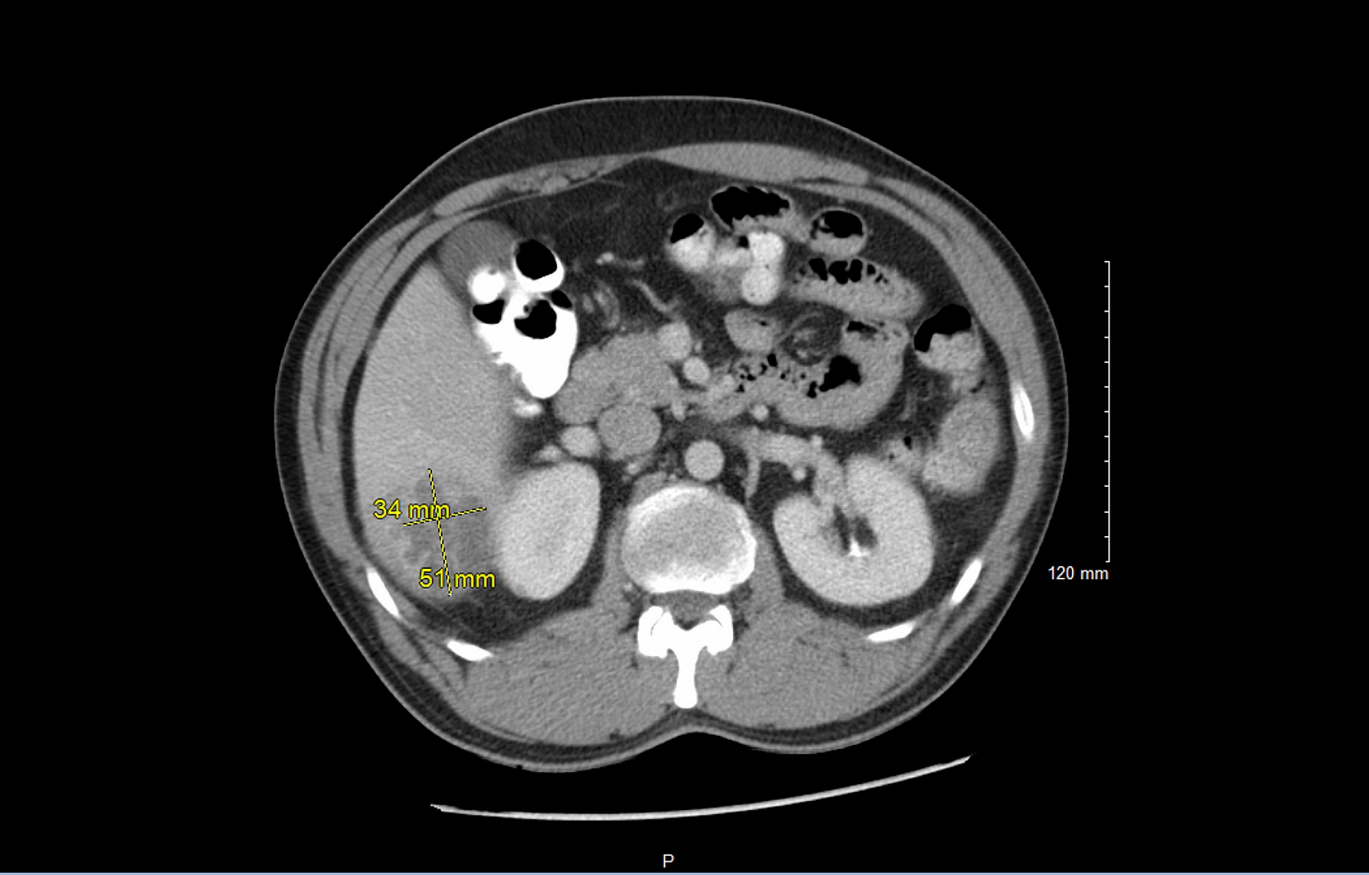
5.1 cm x 3.4 cm x 4 cm multiloculated hepatic abscess in the inferior posterior aspect of the right lobe

A 45-year-old male presented with complaints of right-sided abdominal pain that had been ongoing for four days. He described it as sharp, intermittent, non-radiating, and worse with deep breathing. Past medical history was significant for a 30 pack-year smoking history. Abdominal pain was associated with productive cough. He had initially presented to the emergency department a week prior, primarily with complaints of a productive cough, and was discharged home with the impression of viral illness; however, he returned the following week as his abdominal symptoms progressively worsened. Physical examination revealed tenderness to palpation in the right upper and lower quadrants, with right costovertebral angle tenderness. Laboratory results revealed leukocytosis with a white blood cell (WBC) count of (15.9 x 10(3)/mcL) and increased alanine transaminase (ALT) (63 U/L). Chest x-ray imaging demonstrated signs of atelectasis/consolidation within the right perihilar region. CT abdomen and pelvis with contrast indicated a 5.1 cm x 3.4 cm x 4 cm multiloculated hepatic abscess in the inferior posterior aspect of the right lobe as illustrated in Figure 1.

The patient underwent drainage of the abscess with the insertion of a pigtail catheter by interventional radiology; 34 ml of brown purulent fluid was drained. Fluid cultures came back positive for MRSA. Echinococcus and Entamoeba antibodies were negative. Antibiogram revealed resistance to ampicillin-sulbactam, cefazolin, erythromycin, oxacillin, and penicillin, and sensitivity to clindamycin, daptomycin, gentamicin, linezolid, and vancomycin. The patient was started on intravenous vancomycin 1.5 mg every 12 hours, for a period of three weeks. 

**Figure 2:**
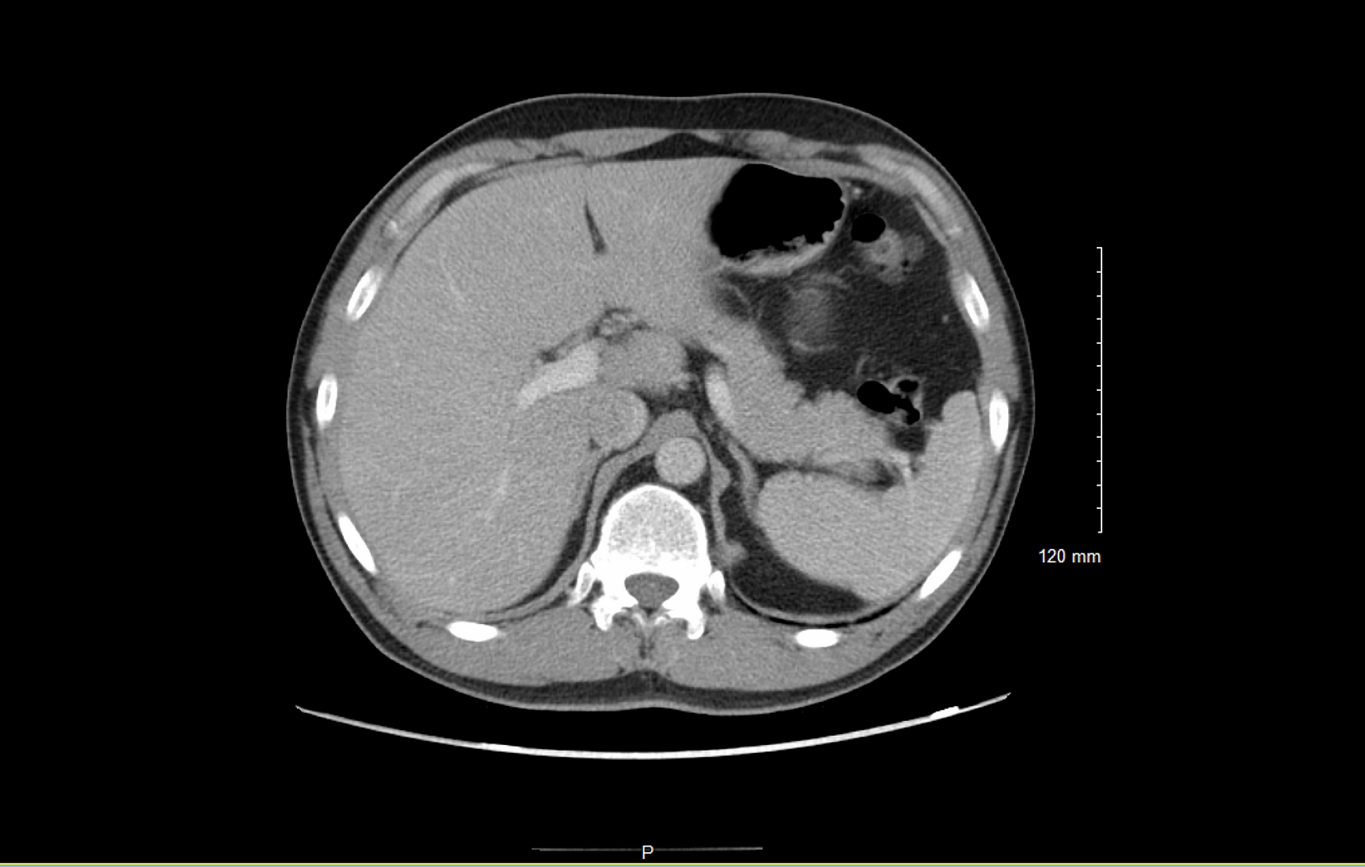
Repeat CT abdomen and pelvis showing resolution of collection with no new abscess

The patient received 18 days of treatment; however, his therapy was interrupted for a period of four days as an outpatient. The patient returned to the emergency department with complaints of right upper quadrant pain for two days. A pigtail catheter was still in place with no drainage. Labs showed resolved leukocytosis with a WBC count of (7.25 mcL), however, liver function tests (LFTs) were elevated; aspartate transaminase (AST) (417 U/L), ALT (1517 U/L), and increased gamma-glutamyl transferase (GGT) (107 U/L). Repeat CT abdomen and pelvis showed a resolved collection with no new abscess (Figure 2). Pigtail catheter was removed.

The patient was re-started on vancomycin therapy, 1.5 mg intravenous, every 12 hours, for four more weeks and ciprofloxacin, 500 mg orally, every 12 hours, for two weeks. LFTs trended downwards and symptoms did not recur.

## Discussion

A liver abscess is described as a pus-filled mass that can be caused by liver injury, or intra-abdominal infection [1]. Pyogenic liver abscesses have an incidence of 0.5%-0.8%, with only 10% of these abscesses being caused by Staphylococcus aureus, and even fewer being caused by MRSA [3]. We present a rare case of a pyogenic liver abscess in which the causative organism was MRSA. 

Clinically, patients will present with symptoms such as right upper quadrant pain (72%), fever (90%), chills (69%), vomiting (32%), and nausea (43%) [7]. Laboratory findings are significant for decreased albumin, increased WBC counts, ALT, AST, and bilirubin [8]. Several imaging modalities such as abdominal X-ray and CT scan may be used to provide further insight into the condition. On imaging, our patient was noted to have a 5.1 cm x 3.4 cm x 4 cm multiloculated hepatic abscess in the inferior posterior aspect of the right lobe. The right lobe of the liver is more commonly affected due to the blood supply insertion predominantly at this location [3]. 

Liver abscesses can be classified as either pyogenic (primarily polymicrobial), or amoebic (primarily Entamoeba histolytica). The majority of pyogenic liver abscesses are caused by the organisms Escherichia coli, Klebsiella, Streptococcus, Staphylococcus, and anaerobes [1]. Fifty percent of the pyogenic causes of liver abscess occur secondary to cholangitis, while less common causes include hepatic artery bacteremia, portal vein bacteremia, diverticulitis, cholecystitis, and penetrating trauma [9]. If a single microbial cause of infection is identified, it is important to determine the source of infection, as the liver abscess most likely occurred through hematogenous dissemination. The major routes of infection of community-acquired MRSA are skin and soft tissue infections, of which around 30% stem from lower limb infections, with hematogenous spread occurring largely secondary to endocarditis and pyelonephritis [1,9] In addition to abdominal pain, our patient presented with a productive cough. Chest X-ray was remarkable for atelectasis and developing consolidation within the right perihilar region. Given these findings, lower respiratory tract infection may have acted as a possible source of infection for the formation of the liver abscess. Community-acquired MRSA often presents as a lower respiratory tract infection [9]. Pneumonia has previously been associated with primary liver abscess [10]. From a review of the literature, there is limited data on MRSA pneumonia leading to liver abscess formation. However, bacteremic pneumonia is considered a potential cause of distal organ abscess formation [9,10]. 

First-line therapy for MRSA infection is intravenous vancomycin [3]. One concern for the presence of MRSA infection is the rising limitations to treatment, as pathogens become increasingly resistant to classic ꞵ lactam drugs. Vancomycin use carries the risk of nephrotoxicity, and there have been reported cases of isolated vancomycin-resistant Staphylococcus aureus [9]. The proposed mechanism of vancomycin resistance is the production of excessive cell-wall peptidoglycan. Linezolid carries a decreased likelihood for the development of new-onset resistance due to its inhibition of protein synthesis early by binding to ribosomal subunits, and is often selected as the drug of choice in vancomycin resistance or if renal contraindications are present [3,9]. Therapeutic intervention includes percutaneous drainage with the administration of empiric antibiotics [1]. If the liver abscess is not treated with antibiotics aggressively for two to three weeks parenterally, followed by two-four weeks orally along with a percutaneous drain, then prognosis may be fatal due to sepsis, peritonitis, or empyema [1,4,6]. The average length of treatment is four-six weeks [11] 

## Conclusions

In conclusion, the current incidence of a hepatic MRSA abscess is rare, but should be considered as a differential in those with risk factors. Understanding the pathophysiology of infection is vital to a successful treatment plan. Allowing for a targeted approach with appropriate antibiotics, therefore, limiting the progression of symptoms and dissemination. 
